# In the fed state, autophagy plays a crucial role in assisting the insect vector *Rhodnius prolixus* mobilize TAG reserves under forced flight activity

**DOI:** 10.3389/fphys.2024.1352766

**Published:** 2024-04-25

**Authors:** Samara Santos-Araujo, Fabio Gomes, Luiz Fernando Carvalho-Kelly, José Roberto Meyer-Fernandes, Katia C. Gondim, Isabela Ramos

**Affiliations:** ^1^ Instituto de Bioquímica Médica Leopoldo de Meis, Universidade Federal do Rio de Janeiro, Rio de Janeiro, Brazil; ^2^ Laboratório de Ultraestrutura Celular Hertha Meyer, Instituto de Biofísica Carlos Chagas Filho, Universidade Federal do Rio de Janeiro, Rio de Janeiro, Brazil

**Keywords:** autophagy, lipophagy, flight activity, Chagas disease, *Rhodnius prolixus*

## Abstract

Autophagy is a cellular degradation pathway mediated by highly conserved autophagy-related genes (Atgs). In our previous work, we showed that inhibiting autophagy under starvation conditions leads to significant physiological changes in the insect vector of Chagas disease *Rhodnius prolixus*; these changes include triacylglycerol (TAG) retention in the fat body, reduced survival and impaired locomotion and flight capabilities. Herein, because it is known that autophagy can be modulated in response to various stimuli, we further investigated the role of autophagy in the fed state, following blood feeding. Interestingly, the primary indicator for the presence of autophagosomes, the lipidated form of Atg8 (Atg8-II), displayed 20%–50% higher autophagic activation in the first 2 weeks after feeding compared to the third week when digestion was complete. Despite the elevated detection of autophagosomes, RNAi-mediated suppression of *RpAtg6* and *RpAtg8* did not cause substantial changes in TAG or protein levels in the fat body or the flight muscle during blood digestion. We also found that knockdown of *RpAtg6* and *RpAtg8* led to modest modulations in the gene expression of essential enzymes involved in lipid metabolism and did not significantly stimulate the expression of the chaperones BiP and PDI, which are the main effectors of the unfolded protein response. These findings indicate that impaired autophagy leads to slight disturbances in lipid metabolism and general cell proteostasis. However, the ability of insects to fly during forced flight until exhaustion was reduced by 60% after knockdown of *RpAtg6* and *RpAtg8*. This change was accompanied by TAG and protein increases as well as decreased ATP levels in the fat body and flight muscle, indicating that autophagy during digestion, i.e., under fed conditions, is necessary to sustain high-performance activity.

## 1 Introduction

*Rhodnius prolixus*, a crucial Chagas disease vector, is classified as an obligatory hematophagous organism and possesses highly specialized metabolic pathways to cope with the high protein contents of ingested blood; through these pathways, other essential macromolecules, including lipids and carbohydrates, are generated (Gondim et al., 2018; Lange et al., 2022). As blood is slowly digested in the days following a blood meal, lipids are absorbed and metabolized by the midgut, secreted into the hemolymph and transported by lipophorin (a major circulating lipoprotein) to the fat body, where they are stored mainly as triacylglycerol (TAG) in lipid droplets (LDs) (Grillo et al., 2007; Pontes et al., 2008; Alves-Bezerra et al., 2017). Amino acids, which are generated in large amounts from the hydrolysis of blood proteins, are used in the fat body for the *de novo* synthesis of lipids, which also accumulate in the fat body (Saraiva et al., 2021; Moraes et al., 2022). Additionally, *R. prolixus* can remain under starvation conditions for relatively long periods of time and can be held in the laboratory with three or more weeks between feedings. During prolonged starvation, energy homeostasis is maintained by accumulated nutrients from the last blood meal; thus, TAG is mobilized from LDs and can be directed to mitochondrial beta-oxidation (Pontes et al., 2008; Gondim et al., 2018; Arêdes et al., 2022; De Paula et al., 2023). Due to its uniqueness, this insect is a fascinating model for research on energy metabolism and physiology, and numerous aspects of its lipid metabolism have been previously investigated (Gondim et al., 1989; Atella et al., 1992; Coelho et al., 1997; Grillo et al., 2007; Pontes et al., 2008; Alves-Bezerra et al., 2010; Santos et al., 2011; Alves-Bezerra and Gondim, 2012; Alves-Bezerra et al., 2016b; Alves-Bezerra et al., 2016a; Alves-Bezerra et al., 2017; Arêdes et al., 2022; Moraes et al., 2022).

Autophagy leads to the intracellular degradation of organelles, which has been canonically characterized as a way of enhancing adaptability to extended periods of starvation and a method to recycle nutrients (Klionsky et al., 2021). Currently, more than 40 autophagy-related genes (Atgs) have been identified in yeast by genetic screening (Tsukada and Ohsumi, 1993; Yao et al., 2015), and most of these genes present clear homologs throughout evolution (Xie et al., 2015; Dikic and Elazar, 2018; Zhou et al., 2020). Among the Atgs, Atg6 is part of a phosphatidylinositol 3-kinase (PI3K) complex that interacts with two proteins related to the intracellular transport of vesicles, Vps15 (vacuolar protein sorting 15) and Vps34 (vacuolar protein sorting 34), as well as another autophagic protein, Atg14. Once this interaction occurs, the complex generates phosphatidylinositol 3–phosphate (PI_3_P). Then, the accumulation of PI_3_P triggers the recruitment of signals necessary for the autophagic process. Atg6 contains a BCL-2 homology domain (BH3), a coiled-coil domain (CCD), and an evolutionarily conserved domain (ECD), which enable multiple interactions with different target proteins (Furuya et al., 2005; Cao and Klionsky, 2007; Devereaux et al., 2013; McKnight and Yue, 2013). Atg8, also known as LC3 (in mammals), is present in the membrane of autophagic organelles and is used as a marker of autophagosomes. This protein occur as LC3-I, a cytosolic form, and LC3-II, a form covalently coupled to phosphatidylethanolamine in the membrane of the growing autophagosome (Kabeya et al., 2000; Nakatogawa, 2013). Atg6 and Atg8 have been extensively studied and are essential for advancing autophagic flux (Cao and Klionsky, 2007; Shpilka et al., 2011).

Various selective forms of autophagy, including mitophagy, ER-phagy, and lipophagy, have been characterized by the reliance on specialized receptors to reach high degrees of specificity in target organelles (Johansen and Lamark, 2011; Rogov et al., 2014). Lipophagy is a type of autophagy that contributes specifically to the degradation of lipids from cytosolic LDs, delivering TAG to the lysosome, providing an alternative pathway for the transfer of fatty acids to mitochondria or other destinations. There is mounting evidence that lipophagy functions as an independent but related mechanism to the long-established pathway of neutral lipolysis, contributing to TAG breakdown (Singh et al., 2009; Cingolani and Czaja, 2016; Sathyanarayan et al., 2017; Zechner et al., 2017).

Most cell types exhibit basal autophagy activity, which may play a housekeeping role in preserving the integrity of intracellular organelles and proteins (Mizushima, 2007). Canonically, autophagy is regulated by nutrient-sensing mechanisms that modulate downstream factors and effectors to allow proper adaptation to cell/organism energy requirements; these effectors include mammalian target of rapamycin (mTOR), AMP-activated protein kinase (AMPK) and peroxisome proliferator-activated receptor alpha (PPARα) (Lin et al., 2013; Lee et al., 2014; Seo et al., 2017; Zhang et al., 2018; Li et al., 2019). The most well-documented trigger for autophagy is regulation by starvation, in which nutritional deprivation acts as an activator of autophagic degradation to recycle macromolecules and maintain energy homeostasis. However, many other autophagy triggers, such as oxidative stress (Filomeni et al., 2015) and hypoxia (Mazure and Pouyssegur, 2010), have been described, and crosstalk with other essential proteostasis pathways, namely, the unfolded protein response (UPR) (Hetz, 2012) and the ubiquitin‒proteasome system (UPS) (Kwon and Ciechanover, 2017; Pohl and Dikic, 2019), has been thoroughly described. As with all degradative pathways, fine-tuned regulation is essential for selectively modulating autophagy; thus, investigating the roles of autophagy under various conditions is crucial for clarifying the intricate details of metabolic adaptation and physiology (Boya et al., 2013).

In previous work, our group investigated the role of autophagy in lipid mobilization under starvation conditions in *R. prolixus*. During starvation, autophagy deficiency triggered TAG retention in the fat body and flight muscle, decreased longevity, reduced spontaneous locomotor activity, reduced forced flight rates, and modulated proteins involved in lipid metabolism. Taken together, these data indicated that autophagy was necessary to mobilize TAG stores, allowing the insect to adapt to poor nutritional conditions (Santos-Araujo et al., 2020). However, it should be noted that the interaction between autophagy and lipid metabolism is a dynamic and intricate process that can be influenced by several variables, such as physiological demands, nutritional state, and environmental changes. Here, we aimed to examine the function of autophagy in the mobilization of lipids in *R. prolixus* in the fed state to further explore the complex role of autophagy in lipid metabolism and its wider physiological consequences in this insect. To investigate this topic, we used RNAi to silence two essential Atgs, Atg6/Beclin1 (*RpAtg6*) and Atg8/LC3 (*RpAtg8*), and examined fat body TAG stores following blood digestion. Greater autophagosome formation was observed over 14 days after blood feeding when the insect was still digesting the last meal and did not reach starvation conditions. Despite the accumulation of autophagosomes, the knockdown of *RpAtg6* and *RpAtg8* did not result in increased TAG stores, and modest changes in the morphology of the fat body LDs were observed. Therefore, despite being active, autophagy is not necessary to mobilize TAG reserves under fed conditions. Interestingly, when the insects were subjected to an assay of forced flight, the knockdown of *RpAtg6* and *RpAtg8* resulted in decreased flight capacity. This change was accompanied by the accumulation of TAG and protein in the flight muscle, suggesting that, in the fed state, autophagy is necessary for the insect to mobilize TAG and protein and thus adapt to stressful conditions of high-performance activity.

## 2 Materials and methods

### 2.1 Bioinformatics

The sequences of *R. prolixus RpAtg6* (RPRC006439) (GAHY01001036.1; from the assembled digestive tract transcriptome) (Vieira et al., 2018) and *RpAtg8* (RPRC014434) (Pereira et al., 2020) were obtained from the *R. prolixus* genome and transcriptome databases available from Vector Base (www.vectorbase.org).

### 2.2 Insects

Insects were maintained at 28°C ± 2°C, a relative humidity of 65%–85%, and 12 h/12 h light/dark cycles. Adult females were used in the experiments and were fed live rabbit blood every 21 days. The technicians working in the animal facility were involved in all aspects of animal care and followed strict guidelines to ensure careful and consistent animal handling. All animal care and experimental protocols were approved by the institutional ethics committee (Comissão de Ética no Uso de Animais—Universidade Federal do Rio de Janeiro CEUA-UFRJ), process 01200.001568/2013-87, order number 149/19.

All females used in this work were obtained from our insectarium, where the insects were fed for the first time (as adult insects) with live-rabbit blood 14 days after the 5th instar nymph to adult ecdysis. After the first blood feeding, the insects (male and female) remained together to mate and generate eggs that hatched as first-instar nymphs to maintain the insectarium. We knew that the females mated because we monitored their oviposition rates and F1 eclosion rates during this cycle (virgin females do not lay many eggs, and the eggs do not hatch because they are not fertilized). After this first blood feeding, all the adult females were fed every 21 days, and only fully gorged insects were used for the experiments (insects are allowed to feed at will and usually gain 6–7 times the initial body weight in 20–30 min). Thus, fully gorged mated females from the second or third blood feeding were used, and they were highly synchronized regarding blood feeding, digestion, and oviposition.

### 2.3 Extraction of RNA and cDNA synthesis

Adult female fat bodies and flight muscles were dissected, grouped (three organs), and homogenized in TRIzol reagent (Invitrogen, Carlsbad, CA, United States) for total RNA extraction. The integrity and quality of the RNA samples were analyzed via electrophoresis on a 2% agarose gel (UBS, Cleveland, OH, United States). The A260/A280 ratios of all the samples were between 1.8 and 2.2. Reverse transcription was carried out using a High-Capacity cDNA Reverse Transcription Kit (Applied Biosystems, Inc., Foster City, United States) with 1 µg of total RNA after RNase-free DNase I (Thermo Fisher Scientific, Waltham, United States) treatment, according to the manufacturer’s protocol.

### 2.4 PCR/quantitative PCR

PCR experiments were performed using the Taq DNA polymerase enzyme (Thermo Fisher Scientific) and specific primers designed to target amplicon sequences of 226 bp and 108 bp for *RpAtg6* and *RpAtg8*, respectively (Table S1). The cycle parameters were as follows: 5 min at 95°C; 35 cycles of 30 s at 95°C, 30 s at 50°C and 1 min at 72°C; and 15 min at 72°C. For qPCR, the reactions were performed using the qPCRBIO 178 SyGreen Mix Separate-ROX Kit (PCR Biosystems Ltd., London, UK) and specific primers designed for the genes of interest (Table S1). Processing was performed in a StepOnePlus™ thermal cycler (Applied Biosystems). The program was 95°C for 10 min; 95°C for 15 s; 60°C for 45 s for 40 cycles; and a dissociation curve. For each sample, the cDNA was diluted 10 times. The Cq values obtained for the blanks were at least ten units above the experimental points. The blanks were created by replacing the amount of cDNA with Milli-Q water. *Rp18S* gene amplification was used for normalization, as previously described (Majerowicz et al., 2011), and its amplification was constant under our experimental conditions, confirming that it was an appropriate endogenous control (Bustin et al., 2009). The relative expression and ΔΔCq values were calculated from the obtained cycle threshold (Cq) values (Livak and Schmittgen, 2001). The same cDNAs were used to verify the gene expression levels of different genes involved in different pathways of lipid metabolism measured by qPCR, as described above.

### 2.5 RNAi knockdown

Double-stranded RNAs (dsRNAs) for the *RpAtg6* and *RpAtg8* genes were synthesized with the MEGAScript RNAi Kit (Ambion, Inc., Austin, United States) using primers already described (Vieira et al., 2018; Pereira et al., 2020), as listed in Table S2. The unrelated bacterial MalE gene (Gene ID: 948538) was used as a control dsRNA (Hansen et al., 2005). One µg of each dsRNA (dsAtg6, dsAtg8, or dsMal) was injected into the hemocoel of adult females using a 10 µL syringe (Hamilton Company, Reno, United States) 18 days after the blood meal (starvation condition), with the same being fed 3 days after the injection. Subsequently, quantifications by qPCR were performed on Days 5 and 10 postfeeding. The effectiveness of the knockdown was verified in each experiment.

### 2.6 Determination of TAG and protein content

The total abdominal ventral fat body and all flight muscles in the thorax were dissected from control and knockdown insects on the 5th, 10th and 14th days after the blood meal. The organs were washed in cold PBS buffer (10 mM sodium phosphate buffer, pH 7.4, 0.15 M NaCl) and individually homogenized in a Potter–Elvehjem tube in 200 μL of cold PBS for the flight muscle and 150 μL for the fat body. The TAG content was determined enzymatically using a Triglycerides 120 kit (Doles Reagents, Goiânia, Brazil). The total protein content was determined according to the methods of Lowry et al. (1951) using bovine serum albumin (BSA) as a standard.

### 2.7 Nile red staining of lipid droplets

Fat bodies were obtained from females treated with dsRNA (at least 3 females of each condition) on the 5th and 10th days after feeding and stained with Nile red and DAPI (Sigma‒Aldrich, Saint Louis, MO, United States), as previously described for LD analysis of *R. prolixus* (Defferrari et al., 2016). Fat bodies were incubated for 15 min in 1 mg/mL Nile red and 2 mg/mL DAPI prepared in 75% glycerol. The tissues were placed on their respective slides in 100% glycerol and immediately photographed on a Leica TCS-SPE laser scanning confocal microscope (Leica Microsystems Suite X). The excitation wavelengths used were 543 nm for Nile red and 280 nm for DAPI. The peripheral regions of the fat bodies were analyzed using a ×20 objective. In three independent experiments, the maximum diameters of LDs were obtained from two images of each group using DAIME image analysis software after automatic edge detection segmentation (Alves-Bezerra et al., 2017). The LD maximum diameters were plotted in frequency histograms (bin width = 2).

### 2.8 Fat body immunostaining of the endoplasmic reticulum

The fat body was obtained from females treated with dsRNA (at least 3 females for each condition) on the 10th day after feeding, followed by fixation with 4% paraformaldehyde for 30 min. After fixation, the organ was incubated in 50 mM ammonium chloride for 1 h and washed in Tris-buffered saline (TBS) (50 mM Tris-HCl, pH 7.5, 150 mM NaCl). The samples were then incubated in a blocking solution of 3% BSA in TBS-T (TBS 0.1% Tween) for 2 h under slow agitation. After an additional wash with TBS, the sections were incubated with primary antibodies against KDEL (1:200; Abcam, cat# ab176333) overnight at 4°C. Then, 3 more TBS washes were performed for 5 min, and the samples were subsequently incubated with the secondary anti-rabbit IgG antibody Alexa Fluor 488 (1:500; Thermo Fisher cat# A32723) for 2 h at room temperature. Finally, the fat bodies were incubated for 15 min in 1 mg/mL Nile red and 2 mg/mL DAPI prepared in 75% glycerol. Tissues were placed on their respective slides submerged in N-propyl gallate and immediately photographed on a Zeiss Elyra confocal microscope in two independent experiments. The fat bodies were analyzed using a ×63 objective with ×2 magnification.

### 2.9 Anti-Atg8 immunoblotting

**FIGURE 1:**
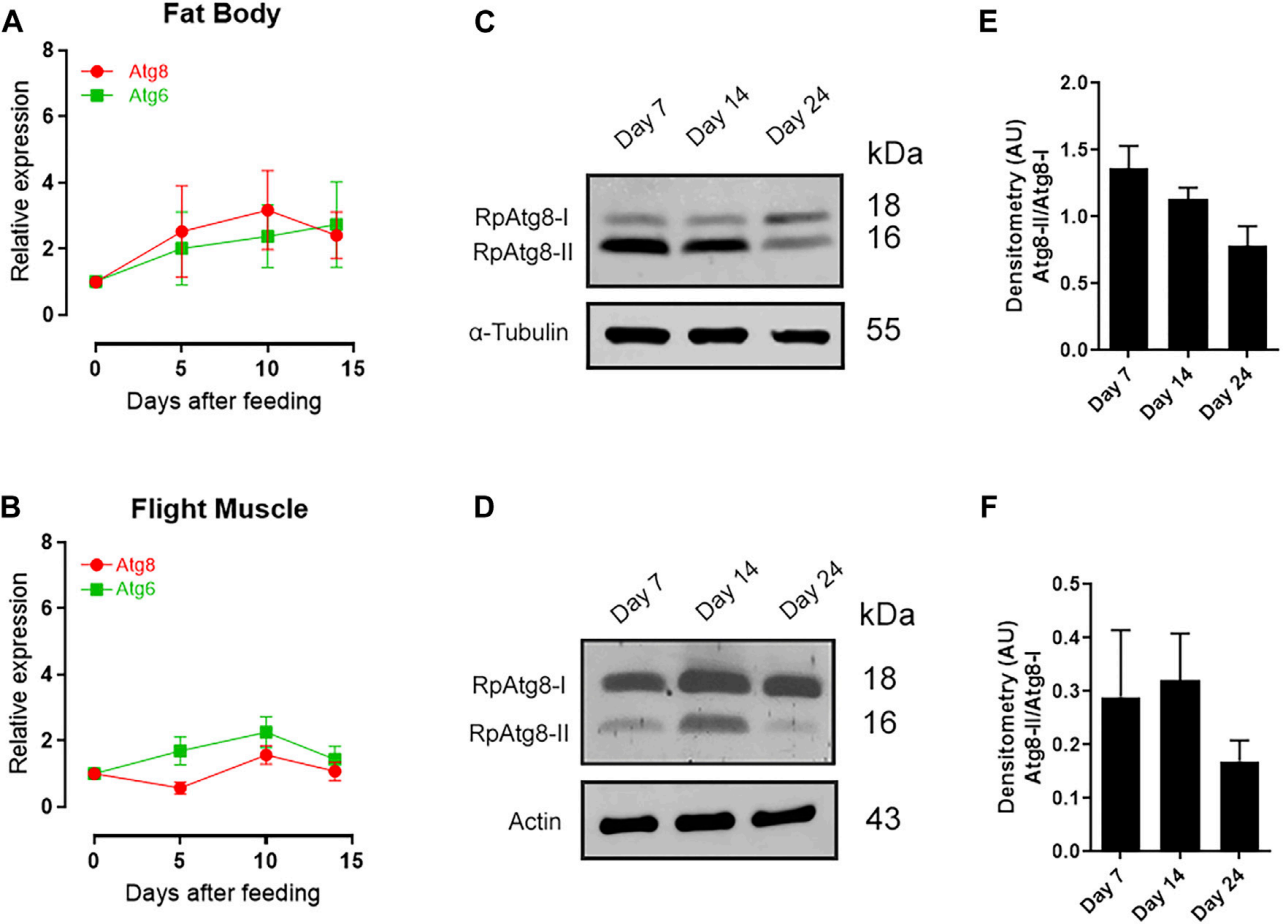
Expression of RpAtg8 and RpAtg6 and autophagosome formation during the reproductive cycle. Adult females were dissected on different days after feeding. *RpAtg8* and *RpAtg6* mRNA levels were determined by qPCR using *Rp18S* expression as a reference gene. **(A)** Quantification of *RpAtg6* and *RpAtg8* mRNA in the fat body. **(B)** Quantification of *RpAtg6* and *RpAtg8* mRNA in the flight muscle. The graphs show the means ± SEMs (*n* = 5–7). **(C)** RpAtg8 immunoblot on the 7th, 14th, and 24th days after feeding in the fat body. **(D)** RpAtg8 immunoblot on the 7th, 14th, and 24th days after feeding in the flight muscle. RpAtg8-I: free RpAtg8; RpAtg8-II: lipidated RpAtg8. **(E** and **F)** RpAtg8-II/RpAtg8-I densitometry on the 7th, 14th, and 24th days after feeding in the fat body and flight muscle, respectively (*n* = 3). p> 0.05 when compared by one-way ANOVA.

**FIGURE 2:**
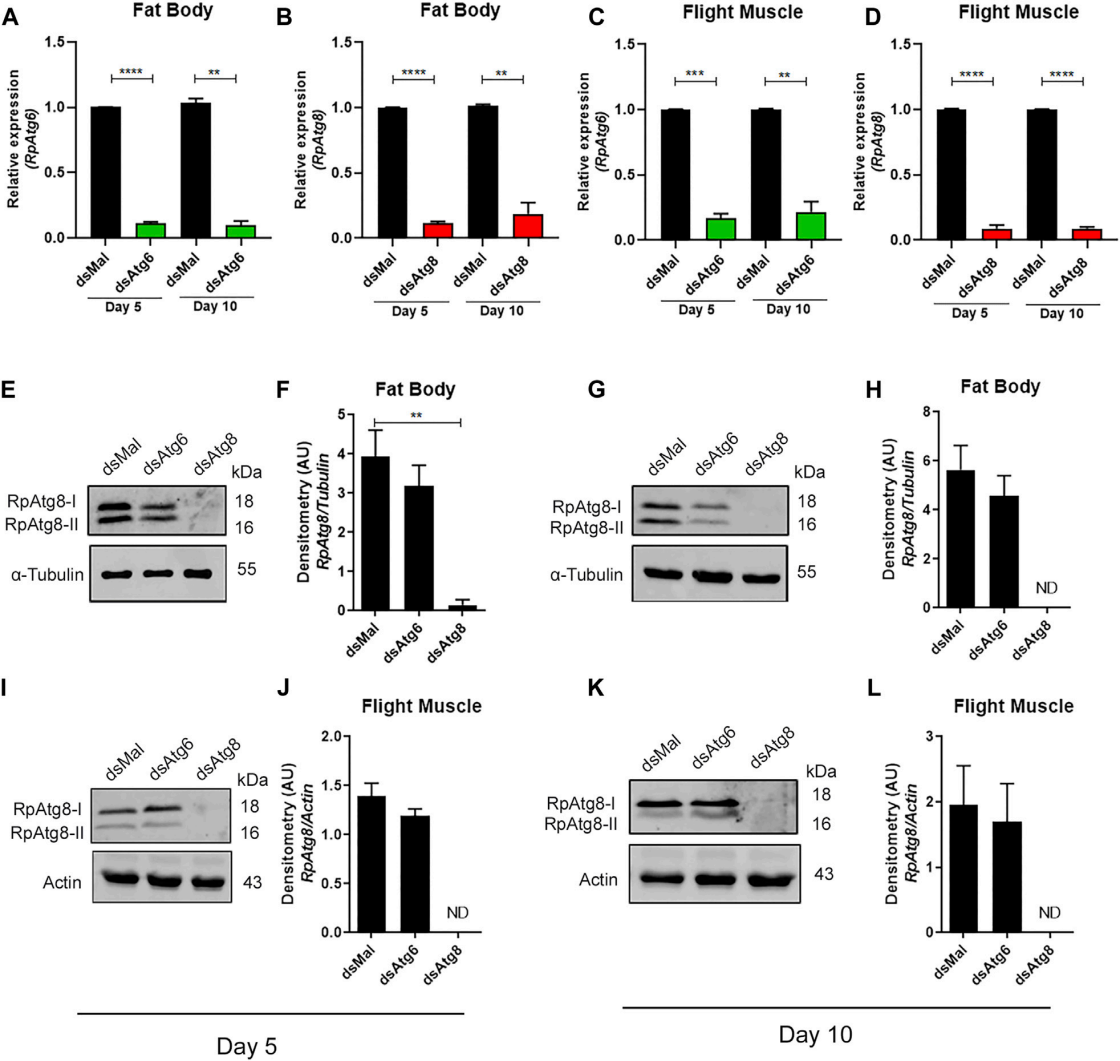
Knockdown of RpAtg6 and RpAtg8 in the fat body and flight muscle. Adult females were injected with 1 µg of dsRNA for *RpAtg6, RpAtg8*, or *Mal* (control) and fed 3 days later; the insects were dissected on the 5th and 10th days after feeding. The mRNA levels were determined by qPCR using *Rp18S* expression as a reference gene. **(A,B)** Quantification of *RpAtg6* and *RpAtg8* mRNA levels in the fat body. **(C,D)** Quantification of *RpAtg6* and *RpAtg8* mRNA in the flight muscle. **(E)** RpAtg8 immunoblot of fat body proteins on the 5^th^ day. The graphs show the means ± SEMs of 4 independent experiments (*n* = 4). **p < 0.01, ***p < 0.001, ****p < 0.0001, compared with Student’s t-test. **(F)** Total RpAtg8 densitometry (RpAtg8-I + RpAtg8-II). **(G)** RpAtg8 immunoblot of fat body proteins on the 10th day. **(H)** Total RpAtg8 densitometry. **(I)** RpAtg8 immunoblot of flight muscle proteins on the 5^th^ day. **(J)** Total RpAtg8 densitometry. **(K)** RpAtg8 immunoblot of flight muscle proteins on the 10th day. **(L)** Total RpAtg8 densitometry. RpAtg8-I: free RpAtg8. RpAtg8-II: lipidated RpAtg8. The graphs show the means ± SEMs of 4 independent experiments (*n* = 4). **p < 0.01, compared by one-way ANOVA followed by Tukey’s *post hoc* test.

Adult females were fed and dissected on different days after feeding (7th, 14th, and 24th days). For the knockdown experiments, adult females were injected on the 18th day after feeding with the respective dsRNAs (dsMal, dsAtg6, or dsAtg8) and fed 3 days later. Dissections were carried out on the 5th and 10th days after feeding. The fat bodies (pools of three organs) were homogenized in a Potter-Elvehjem tube in 100 μL of cold PBS, while the flight muscles were homogenized in 200 μL of PBS; however, the samples were homogenized individually in this case. For the experiments in which autophagosomes were detected on different days after the blood meal (Figure 1), aliquots containing 25 µg of total protein were used. To verify the RpAtg8 protein levels in the control and knockdown groups (Figure 2), 60 µg of total protein was used. All the samples were separated via 13% SDS‒PAGE, and the proteins were subsequently transferred to nitrocellulose membranes (GE Healthcare Life Sciences, USA). The membranes were blocked in blocking buffer (10 mM Tris, pH 7.5; 0.15 M NaCl, 0.1% Tween-20, and 5% skim milk) for 1 h and incubated overnight at 4°C with antibodies raised in rabbits (1:2,500 in blocking buffer) against *R. prolixus* RpAtg8 (Pereira et al., 2020). The membranes were washed 3 times for 10 min with blocking buffer and then incubated with the secondary antibody (goat anti-rabbit-horseradish peroxidase (HRP) conjugate Ab6721; Abcam, Cambridge, MA, United States) diluted 1:20,000 for 1 h. Two different primary antibodies were used for the loading controls. Rabbit polyclonal anti-tubulin (#2144 Cell Signaling Technology, Danvers, MA, United States) for the fat body (1:5,000) and anti-β actin (Santa Cruz Biotechnology, Santa Cruz, CA, United States) for the flight muscle (1:1,000) were incubated overnight at 4°C. For tubulin, the secondary antibody used was diluted 1:20,000 for 1 h of incubation (Ab6721, goat anti-rabbit-HRP conjugate, Abcam). For β-actin, the same secondary goat anti-mouse HRP diluted 1:3,000 (Ab6789, Abcam) was used with the same incubation time. After the membranes were washed with blocking buffer, they were developed with an enhanced chemiluminescence (ECL) system (2.5 mM luminol in dimethyl sulfoxide (DMSO), 0.4 mM coumaric acid, 0.02% hydrogen peroxide in water and 0.02% 1 M Tris, pH 8.4) for 1 min. The intensity of the bands was analyzed via densitometry with ImageJ software version 1.50i (NIH Image, Bethesda, MD, United States) with background corrections.

### 2.10 Assay for forced flight activity

Females treated with dsRNA on the 18th day after feeding were fed 3 days later and subjected to a forced flight test on the 10th day after the blood meal (Weis-Fogh, 1956; Oliveira et al., 2006). Briefly, the insects were hung by a thread, which was attached to the dorsal surface of the thorax. A fan was used to generate continuous airflow and the insects flew until exhaustion. Insects were considered to be exhausted when they stopped flying for more than 30 s despite continuous stimulation by airflow. The duration of flight was recorded for each insect.

### 2.11 Intracellular ATP quantification

The intracellular ATP concentration was quantified using an ATP bioluminescent somatic cell assay kit (Sigma‒Aldrich). Briefly, fat bodies and flight muscles were obtained (10 days after the blood meal) and homogenized in PBS (100 µL), after which the intracellular ATP content was determined using an adenosine 5′- triphosphate (ATP) bioluminescent somatic cell assay kit (Sigma‒Aldrich) and an ATP standard curve, which was prepared for each experiment (Carvalho-Kelly et al., 2020; Almeida-Oliveira et al., 2023). The final results were normalized to the amount of protein in the samples.

### 2.12 Statistics

Relative expression and ΔΔCq values were calculated from the obtained cycle threshold (Cq) values and were used for statistical analyses. Relative expression values (2^−ΔΔCq^) were used for data plotting only. The results were analyzed by Student’s t-test to compare two different conditions and one-way ANOVA followed by Tukey’s test to compare more than two conditions. TAG and protein levels in control and knockdown insects were measured on different days after the blood meal and were analyzed using two-way ANOVA followed by Tukey’s test. A Kruskal‒Wallis test followed by Dunn’s test was used to compare LD diameters. To compare the LD frequency distributions among the histograms, the raw values were used, and chi-square tests were performed to compare the observed distributions against the expected distributions. Differences were considered significant at *p* < 0.05, and all the statistical analyses were performed using Prism 8.0 software (GraphPad Software, San Diego, CA, United States).

## 3 Results

### 3.1 *RpAtg6* and *RpAtg8* are expressed in the fat body and flight muscle throughout the reproductive cycle

In hematophagous insects, such as *R. prolixus*, gene expression patterns in different organs, including genes related to lipid metabolism, are commonly associated with blood feeding (Gondim et al., 2018; Leyria et al., 2020). Thus, we aimed to investigate whether autophagy-related genes are regulated by the blood meal in *R. prolixus*. Females were dissected at various time points during the gonadotrophic cycle to quantify the gene expression of *RpAtg6* and *RpAtg8*. Specifically, we conducted this experiment before feeding (Day 0) and on the 5th, 10th, and 14th days after the blood meal (Figures 1A,B). We observed that for the fat body (Figure 1A) and for the flight muscle (Figure 1B), statistically significant differences could not be detected in the gene expression of either gene over the chosen days; therefore, demonstrating these genes are not modulated in response to the nutritional status of the insect.

Since the gene expression was not altered, it was also important to analyze whether these genes were modulated at the protein level. For this purpose, immunoblotting was carried out using an antibody against Atg8, which was divided into its free form (RpAtg8-I) or its lipidated form (RpAtg8-II), the latter being an important marker of autophagosomes. The aim of this experiment was to determine whether the presence of autophagosomes was more pronounced on certain days in our study. Our findings indicated that the levels of lipidated RpAtg8 (RpAtg8-II) tended to decrease during starvation, specifically on the 24th day, compared to the preceding days (7th and 14th days). This trend was observed in the fat body (Figures 1C, E) and the flight muscle (Figures 1D, F). While this result did not yield a statistically significant difference, it suggests a higher autophagic activity during the initial days following the blood feeding than under starvation conditions (24 days after feeding).

### 3.2 RNAi-mediated knockdown of *RpAtg6* and *RpAtg8* was effective in the fat body and flight muscle throughout the reproductive cycle

On the 18th day after feeding, adult females were injected with specific double-stranded RNAs designed to target the *RpAtg6* and *RpAtg8* sequences of *R. prolixus*. Three days after dsRNA injection, the females were fed and dissected 5 and 10 days after feeding. After analysis via qPCR, we found that mRNA knockdown was efficient for both genes, with at least 80% knockdown in the fat body and flight muscle (Figures 2A–D). To test whether inhibiting gene expression could also result in reduced protein levels and impair autophagosome biogenesis, we performed immunoblotting and found that total RpAtg8 levels (free and lipidated, RpAtg8-I and RpAtg8-II, respectively) were markedly reduced in fat body and flight muscle knockdowns for *RpAtg8* on the 5th and 10th days after feeding (Figures 2E–L). These findings indicate that autophagosome biogenesis was impaired in these organs. In contrast, knockdown of *RpAtg6* did not induce any changes in RpAtg8 protein levels (Figures 2E–L).

### 3.3 *RpAtg6* and *RpAtg8* knockdown does not alter the levels of TAG or protein in the fat body or flight muscle during blood digestion

The fat body is the main organ involved in the storage and metabolism of lipids in insects. Therefore, we investigated the participation of autophagy in the mechanism of TAG mobilization in this organ. The flight muscle also stores significant amounts of TAG within LDs, which is a unique trait of some Hemiptera, including *R. prolixus* (Ward et al., 1982). For this reason, we evaluated TAG levels in flight muscles of insects after knockdown. Three days after dsRNA injection, the females were fed and dissected on days 5, 10, and 14 after feeding. For each dissected day, the levels of TAG and protein were measured in each organ.

**FIGURE 3:**
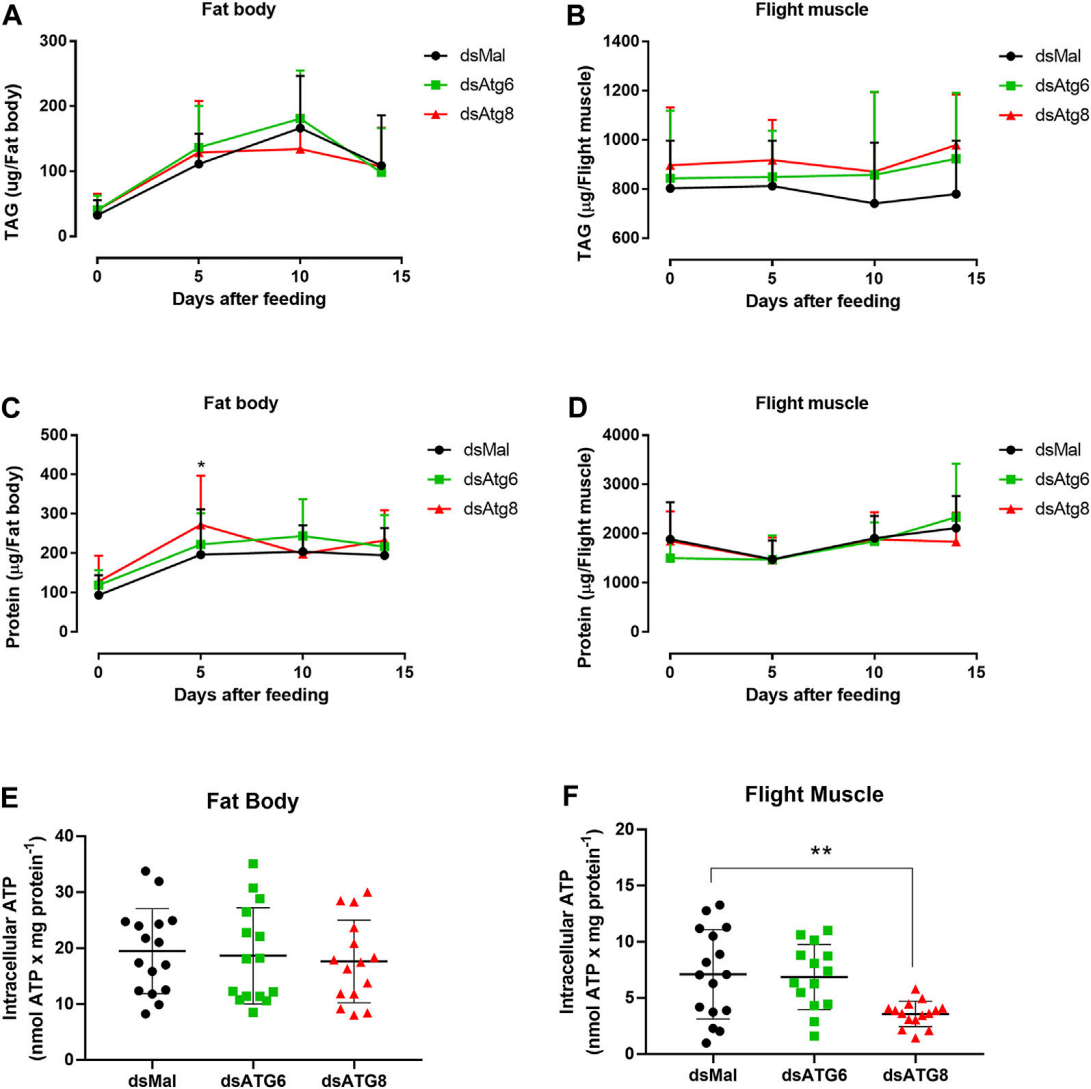
Females knockdown for *RpAtg6* and *RpAtg8* did not show changes in TAG content in the fat body and flight muscle. Adult females were injected with 1 µg of dsRNA for *RpAtg6*, *RpAtg8*, or *Mal* (control) and fed 3 days later; the insects were dissected on different days after feeding. Fat bodies and flight muscles were obtained, washed, and individually homogenized, after which the total amounts of TAG **(A** and **B)** and protein **(C** and **D)** were determined. The graphs show the means ± SDs (*n* = 12–21) *p < 0.05 (Day 5, dsMal × dsAtg8) compared with two-way ANOVA followed by Tukey’s *post hoc* test. The total abdominal ventral fat body and all the flight muscles in the thorax were dissected, and the results are shown as µg per organ. **(E** and **F)** Intracellular ATP levels were quantified in the fat body and flight muscle 10 days after the blood meal. The graphs show the means ± SDs (*n* = 8–21 females; obtained from 4 independent experiments). **p < 0.01, compared by one-way ANOVA followed by Tukey’s *post hoc* test.

High individual variations in TAG and protein levels were observed among the insects since all measurements were performed individually, not in pools. Interestingly, neither *RpAtg6* nor *RpAtg8* knockdown affected the dynamics of TAG accumulation and mobilization in the fat body or the amount of TAG present in this organ on the different days after the blood meal (Figure 3A). Similarly, no differences in TAG content were observed in the flight muscle of the knockdown females (Figure 3B). Regarding the amount of total protein, the fat body of the insects injected with dsAtg8 significantly increased on Day 5 (compared to that of the dsMal), while no alterations were observed on the other analyzed days (Figure 3C). No differences were observed in the levels of proteins in the flight muscle (Figure 3D) between the control (dsMal) and knockdown insects. Interestingly, the content of intracellular ATP was significantly decreased only in the flight muscles of *RpAtg8* knockdown samples (Figures 3E,F).

### 3.4 *RpAtg6* and *RpAtg8* knockdown alters the morphology of LDs and the mRNA levels of lipid metabolism enzymes during blood digestion

**FIGURE 4:**
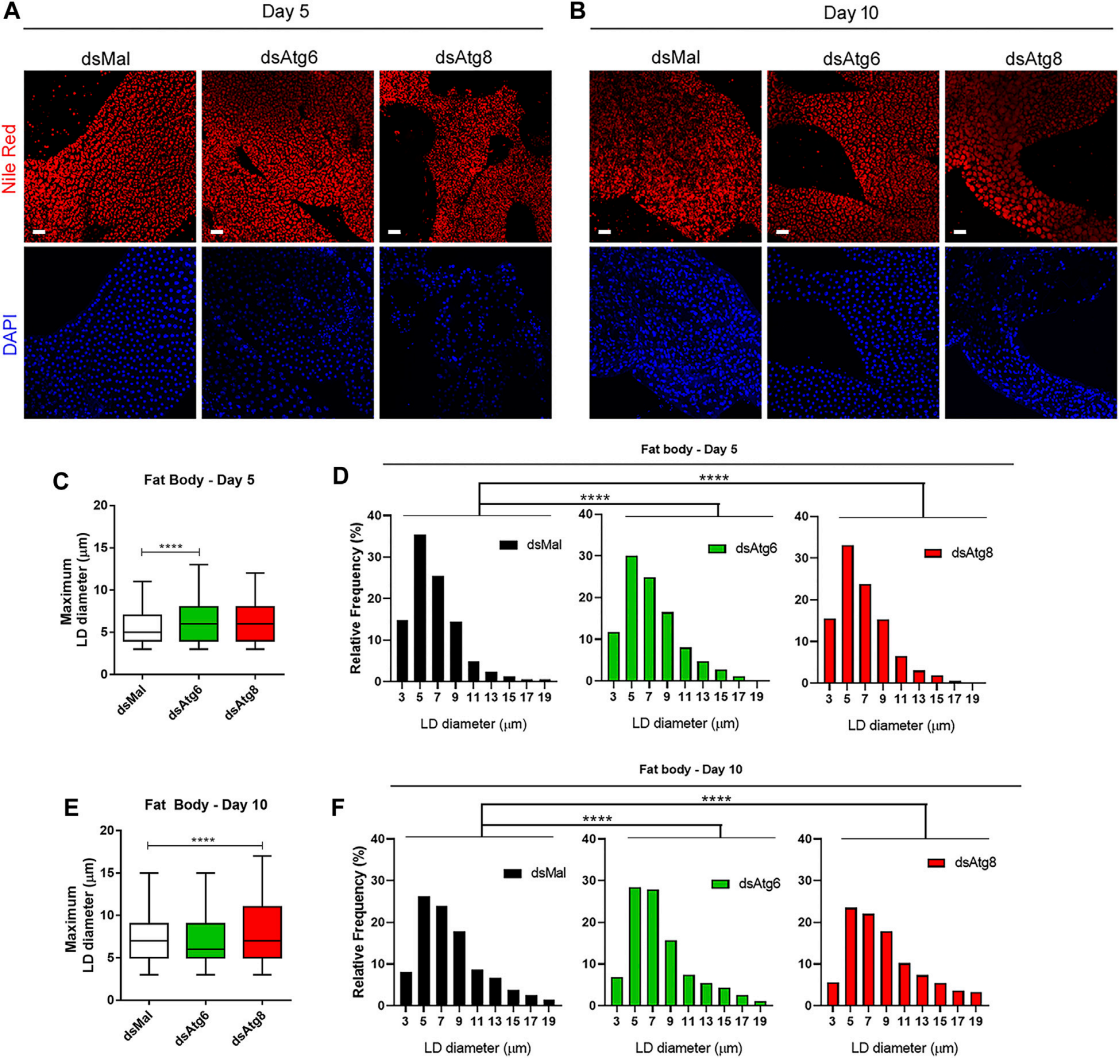
Females knockdown for *RpAtg6* and *RpAtg8* display alterations in the diameters of lipid droplets in the fat body. Adult females were injected with 1 µg of dsRNA for *RpAtg6*, *RpAtg8*, or *Mal* (control) and fed 3 days later. **(A)** Lipid droplets (LDs) in freshly dissected fat bodies on the 5th day or **(B)** the 10th day after feeding from control and knockdown females, stained with Nile Red and observed under a confocal laser scanning microscope. DAPI-stained nuclei were also observed. Bars: 40 µm. **(C)** Quantification of the maximum diameters of the LDs on the 5th day after feeding. Three experiments were performed, and 2 images from each experiment were quantified. **(E)** Quantification on the 10th day after feeding. The graphs show the medians ± 5th-95th percentiles of at least 2000 LDs per condition. ****p < 0.0001, when compared by the Kruskal‒Wallis test followed by Dunn’s *post hoc* test. **(D** and **F)** Histograms of the LD diameter distribution. ****p < 0.0001, when compared by chi-square test.

The number and size of LDs are affected by the rates of lipolytic and lipogenic activities (Schulze et al., 2017; Zechner et al., 2017). Thus, we wondered whether the overall size of LDs could be affected in females with autophagy deficiency. To address this question, we incubated fat bodies with Nile red on the 5th and 10th days after feeding (Figures 4A, B) and quantified the size of the observed LDs (Figures 4C–F). When analyzing the LDs, we found that, despite the sizeable internal variation in the maximum diameters of the LDs, *RpAtg6* knockdown resulted in statistically larger LDs in the fat body on the 5th day (Figure 4C) when compared to the control, and both knockdowns resulted in statistically different distributions of LDs, with higher proportions of the LDs ranging from 9 to 17 µm (Figure 4D). In contrast, on the 10th day, the knockdown of *RpAtg8* generated statistically larger LDs (Figure 4E) with higher proportion of LDs ranging from 11 to 19 μm, whereas knockdown of *RpAtg6* resulted in higher proportions of 5–7 µm LDs, when compared to the control group (dsMal) (Figure 4F).

To test whether the knockdown of *RpAtg6* and *RpAtg8* led to changes in the expression levels of proteins related to lipid metabolism in the fat body and flight muscle, we measured the expression of the following genes, which are essential for these pathways: the lipase brummer (Bmm), the ATGL ortholog in insects; acetyl-CoA-carboxylase (ACC); the long–chain acyl-CoA synthetase 2 (ACSL2); the adipokinetic hormone receptor (AKHr); carnitine palmitoyltransferase 1 (CPT1); diacylglycerol acyltransferase 1 and 2 (DGAT1 and 2); and glycerol–3– phosphate acyltransferase 1 and 4 (GPAT1 and 4).

**FIGURE 5:**
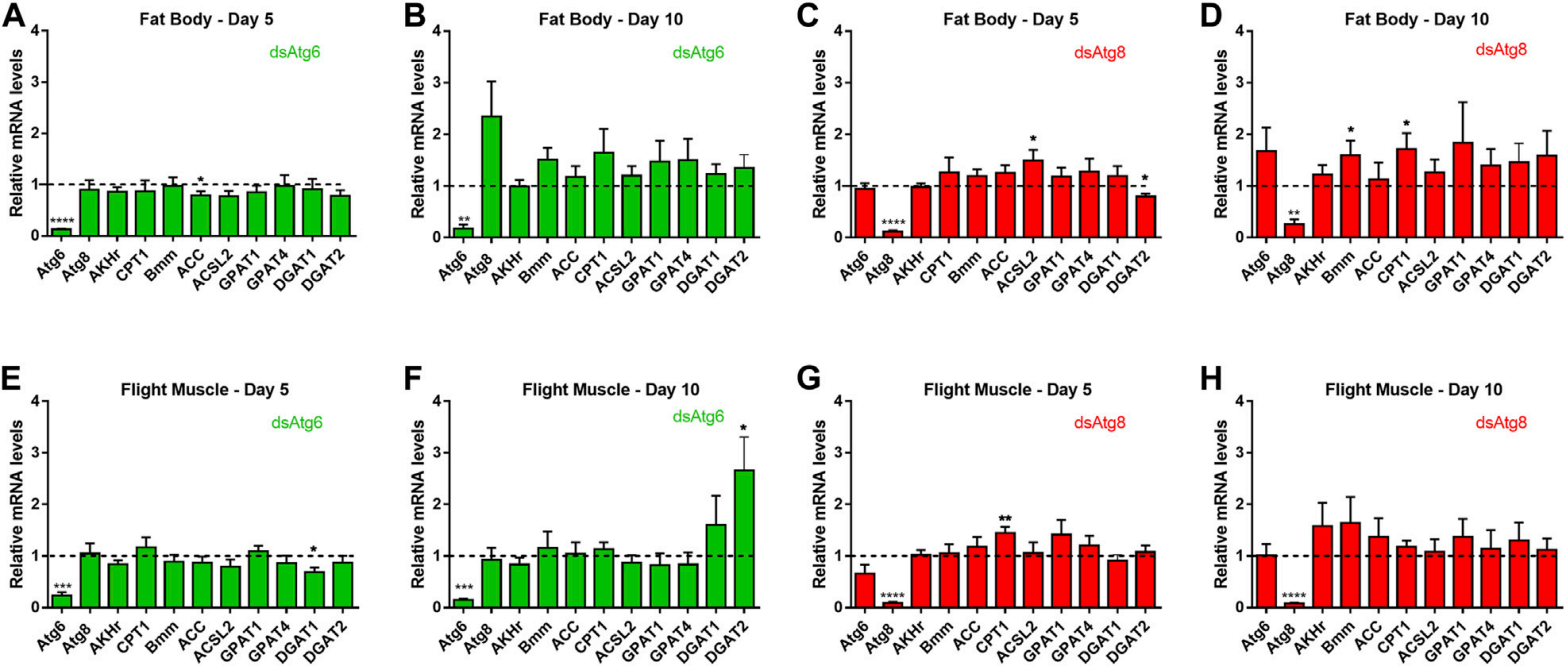
Knockdown of *RpAtg6* and *RpAtg8* slightly affects the expression of genes related to lipid metabolism. Adult females were injected with 1 µg of dsRNA for *RpAtg6*, *RpAtg8*, or *Mal* (control) and fed 3 days later. **(A–D)** Gene expression levels in the fat body were determined by qPCR using specific primers designed for target genes. **(E–H)** The same procedure was used for the flight muscle. *Rp18S* amplification was used as an endogenous control. Gene expression levels are relative to each control value (dashed lines). The graphs show the means ± SEMs of 4 independent determinations, *n* = 4. *p < 0.05, **p < 0.01, ***p < 0.001, ****p < 0.0001, compared by Student’s t-test. AKHr, adipokinetic hormone receptor; Bmm, Brummer lipase; CPT1, carnitine palmitoyltransferase I; GPAT1, glycerol-3-phosphate acyltransferase 1; GPAT4, glycerol-3-phosphate acyltransferase 4; ACSL2, long chain acyl-CoA synthetase 2; ACC, acetyl-CoA carboxylase; DGAT1, diacylglycerol acyltransferase 1; DGAT2, diacylglycerol acyltransferase 2.

In the fat body, only the enzymes ACSL2, Bmm and CPT1 displayed moderate but statistically significant differences in upregulation even though knockdown of *RpAtg6* and *RpAtg8* was effective; in contrast, the enzymes ACC and DGAT2 were downregulated by approximately 20% (Figures 5A–D). In the flight muscle, DGAT2 and CPT1 were upregulated, while DGAT1 was downregulated (Figures 5E–H), suggesting that deficient autophagy had a modest impact on overall lipid metabolism in both organs.

### 3.5 Autophagy-deficient females exhibit discrete alterations in the expression levels of enzymes related to the unfolded protein response and changes in the morphology of the ER in the fat body.

**FIGURE 6:**
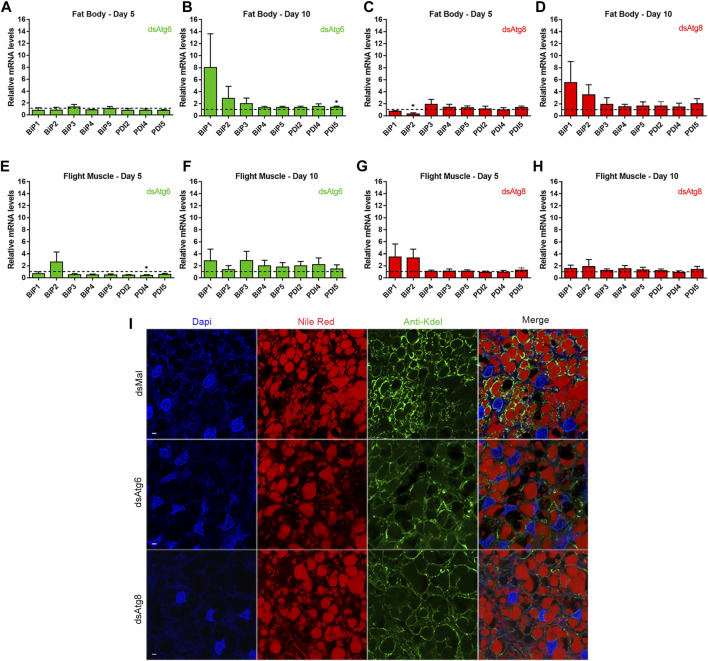
Females knockdown for *RpAtg6* and *RpAtg8* present changes in the ER morphology in the fat body. Adult females were injected with 1 µg of dsRNA for *RpAtg6*, *RpAtg8*, or *Mal* (control) and fed 3 days later. **(A–D)** Expression levels of the ER chaperones BiPs and PDIs in the fat body of knockdown females determined by qPCR. **(E–H)** Expression levels of the ER chaperones BiPs and PDIs in the flight muscle of knockdown females determined by qPCR. *Rp18S* amplification was used as an endogenous control. Gene expression levels are relative to each control value (dashed lines). The graphs show the means ± SEMs of 4 independent determinations, *n* = 4. *p < 0.05, when compared by Student’s t-test. **(I)** Dissected fat bodies on the 10th day after feeding of control or knockdown females were fixed with 4% paraformaldehyde, stained with anti-Kdel and Nile Red, and observed under a confocal laser scanning microscope. DAPI-stained nuclei were also observed. Bars: 40 µm.

Even small changes in autophagy levels can affect protein homeostasis and cause the UPR to be activated, allowing cells to adapt to various environmental conditions (Hetz, 2012). Therefore, we investigated whether the UPR-responsive ER chaperones protein disulfide isomerase (PDI) (Wang, 1998; Wang et al., 2015; Perri et al., 2016) and immunoglobulin heavy-chain-binding protein (BiP) (Pobre et al., 2019) would be altered in our knockdown samples. A few changes were observed after the knockdowns were implemented (Figures 6A–H). Interestingly, statistically significant decreases in BiP2 and PDI4 were detected on Day 5 after *RpAtg8* was knocked down in the fat body (Figure 6C) and after *RpAtg6* was knocked down in the flight muscle (Figure 6E). Among PDIs, only PDI5 displayed a slight 10%, but significant, upregulation at day 10 in the fat body of *RpAtg6*-knockdown samples (Figure 6B). To analyze additional markers of UPR activation, we performed an immunostaining assay using antibodies against the ER-retention signal KDEL to investigate possible changes in ER morphology. The fat bodies of females 10 days after feeding were used for this test. We found that the knockdown of *RpAtg6* and *RpAtg8* resulted in slight changes in the general morphology of the ER, in which control tissues displayed punctate brighter signals, while knockdown samples presented more dispersed staining around the LDs (Figure 6I).

### 3.6 *RpAtg6* and *RpAtg8* knockdown insects display impaired forced flight capacity

**FIGURE 7:**
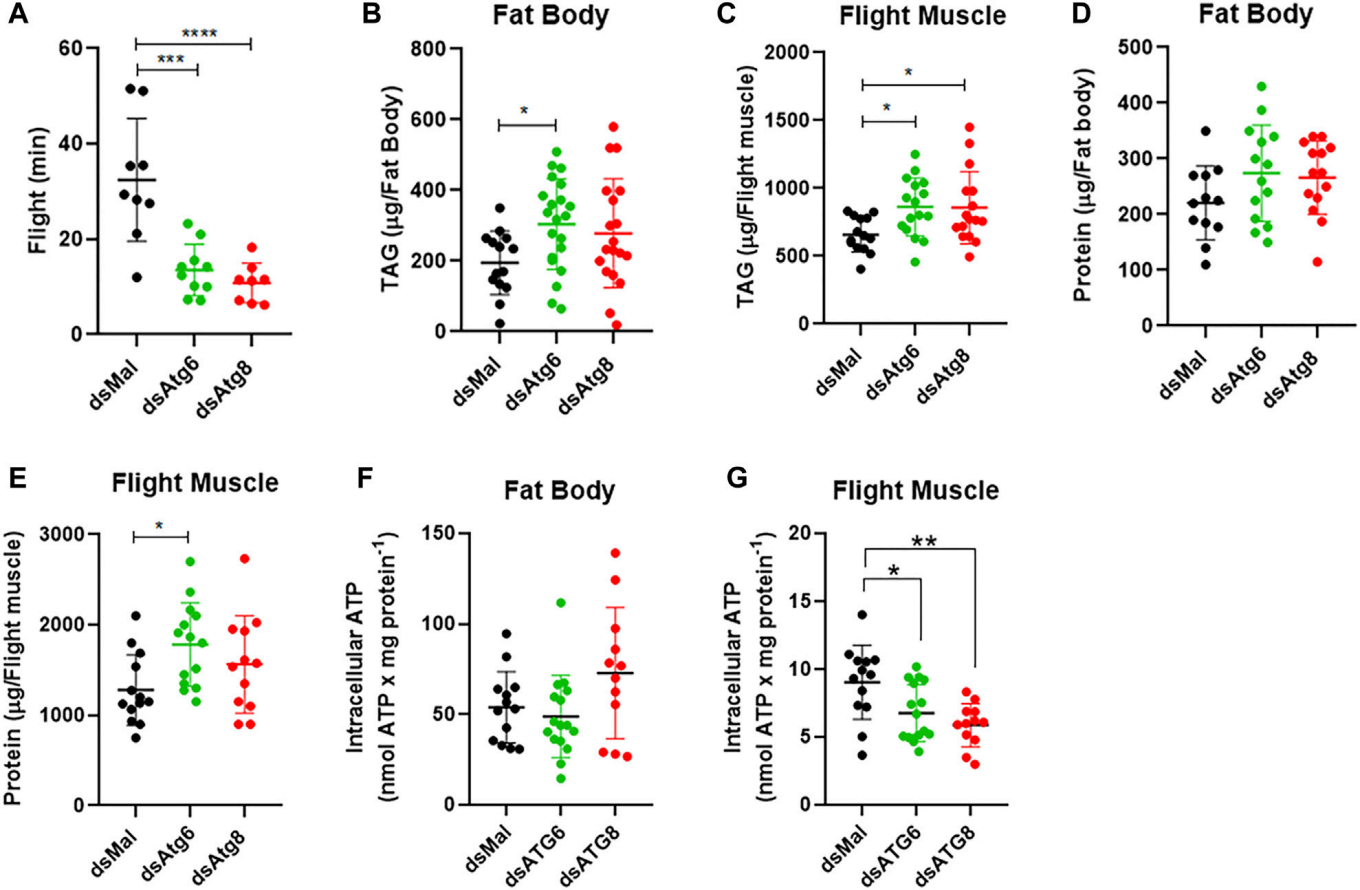
Females knockdown for *RpAtg6* and *RpAtg8* exhibit reduced flight capacity accompanied by reduced ATP levels. Adult females were injected with 1 µg of dsRNA for *RpAtg6*, *RpAtg8*, or *Mal* (control). Three days later, the animals were fed and subjected to a forced flight test on the 10th day after feeding. **(A)** The duration of flight activity until exhaustion was individually recorded. *n* = 9 (dsMal), *n* = 10 (dsAtg6) and *n* = 8 (dsAtg8). After the forced flight, the females were dissected, and TAG levels were quantified in **(B)** the fat body and **(C)** the flight muscle. The total protein levels after the flight were also quantified in the fat body **(D)** and the flight muscle **(E)**. **(F–G)** Intracellular ATP levels in the fat body and flight muscle were quantified after the forced flight assays. The graphs show the means ± SDs. *p < 0.05, **p < 0.01, ***p < 0.001, ****p < 0.0001 when compared by one-way ANOVA followed by Tukey’s *post hoc* test.

To further investigate whether the knockdown of *RpAtg6* and *RpAtg8* results in changes in flight physiology, the insects were subjected to high-performance activity in an assay of forced flight. In this experiment, the insects were hung individually by a thread in front of a tube with continuous airflow and were forced to fly until complete exhaustion. *RpAtg6* and *RpAtg8* knockdown markedly decreased the forced flight capacity of the insects, as the flight time decreased from an average of 35 min with the control insects to 10–15 min with the knockdown insects (Figure 7A). After periods of forced flight, TAG and protein levels were measured in the fat body and flight muscle of each insect to investigate whether this decrease in flight ability could result from a decreased use of energy reserves.

Although the individual variations in TAG and protein measurements are high, statistically significant differences were observed for TAG levels in the fat body after the knockdown of *RpAtg6* and flight muscle after both knockdowns (Figures 7B,C), as well as larger amounts of protein in the flight muscle after the knockdown of *RpAtg6* (Figures 7D,E). In addition, TAG retention in the flight muscle was accompanied by reduced levels of intracellular ATP (Figure 7G). These results suggest that, in the fed state, autophagy is necessary for mobilizing TAG stores during high-performance exercise.

## 4 Discussion

Autophagy functions at basal levels in virtually all cell types under favorable growth conditions. Still, it can also be massively induced by a wide range of environmental and developmental stimuli, including nutrient starvation, senescence, pathogens, metabolic stress, and many other abiotic and biotic stimuli (Levine and Kroemer, 2008; Jaishy and Abel, 2016; Anding and Baehrecke, 2017; Wang et al., 2017; Nakamura and Yoshimori, 2018; Fan et al., 2019). The most well-described mechanism is the regulation of autophagy by starvation; that is, nutritional deprivation activates the degradation process by autophagy, aiming to maintain energy homeostasis. However, little is known about the role of autophagy activity in the fed state, especially regarding lipophagy in insects.

Unexpectedly, we discovered that the levels of the lipidated protein RpAtg8 tended to be higher on the 7th and 14th days following feeding rather than at the expected starvation period (24th day following feeding), which suggests that the time when autophagy is most active in the fat body and flight muscle is when digestion is still occurring, under fed conditions. According to the most recent research, in addition to canonical starvation adaptation, autophagy performs more physiological and pathophysiological tasks than previously believed, including development, anti-aging, microbial eradication, cell death, tumor suppression, antigen presentation, and intracellular protein and organelle clearance (Mizushima, 2007).

In eukaryotic cells, proteostasis is achieved through a fine-tuned balance between synthesis and degradation pathways. During blood digestion, the fat body undergoes vitellogenesis. Under these conditions, the cells in the fat body are highly secretive and likely require dedicated regulation of degradative pathways to counterbalance high synthesis rates (Roy et al., 2018). Autophagy in the fat body may be dedicated to clearing the load of yolk protein precursor synthesis to support sustained vitellogenesis activity. In mosquitoes, it was suggested that an increase in autophagy at the end of vitellogenesis in the fat body is necessary to allow the clearance of the yolk protein precursor vitellogenin for the termination of vitellogenesis and the beginning of a new gonadotrophic cycle (Bryant and Raikhel, 2011).

Under in-house conditions, i.e., where the insects are maintained under controlled and favorable conditions of temperature, light and dark cycles, humidity, and free locomotion inside their containers, the knockdown of *RpAtg6* and *RpAtg8* did not trigger changes in the accumulation of TAG or protein in the fat body or in the flight muscle. Therefore, in this setting, autophagy is not needed to mobilize energy stores. However, a few moderate changes were observed in the fat body, such as changes in the morphology of LDs, the expression of lipid metabolism-related enzymes, and the expression of markers of UPR activation. Therefore, impaired autophagy triggered adaptations that most likely allowed the cells to accomplish general physiological tasks as efficiently as control individuals.

Following a blood meal in *R. prolixus*, fat body cells synthesize lipids and incorporate lipids from the hemolymph to store them as TAG in LDs (Pontes et al., 2008; Saraiva et al., 2021; Silva-Oliveira et al., 2021). Moreover, at the same time, lipids are secreted from the fat body to the circulating lipophorin (Coelho et al., 1997).This finding indicates that lipid metabolism in the fat body is highly active via synthetic and degradative pathways. Although both processes occur, the amount of TAG in the fat body increases after a blood meal, and *RpAtg6* and *RpAtg8* knockdown during the fed state had no effect on TAG content, in contrast to the events observed during starvation, when the TAG content was extremely low and increased following *RpAtg6* and *RpAtg8* knockdown (Santos-Araujo et al., 2020). One possibility is that, in fed insects, lipid synthesis is counterbalanced by the upregulation of degradative pathways, as observed when the expression of other lipid metabolism genes was modulated. In *R. prolixus*, ACSL2 was shown to activate fatty acids that are directed to mitochondrial beta-oxidation (Alves-Bezerra et al., 2016b), and its gene expression increased after *RpAtg8* knockdown. Similarly, the gene expression of CPT1, which is involved in the transport of activated fatty acids inside mitochondria (De Paula et al., 2023), also increased. The same result was observed for Bmm gene expression (Arêdes et al., 2024), which could result in an increase in neutral lipolysis. Moreover, the transcript levels of ACC and DGAT2, genes involved in lipid synthesis (Alves-Bezerra and Gondim, 2012; Saraiva et al., 2021), were reduced. Thus, the coordinated modulation of the expression of these genes may have helped control lipid storage in the fat body of the knockdown insects, resulting in no changes in TAG stores.

Regarding LDs, organelles with larger diameters were observed on the 5th day after *RpAtg6* knockdown; on the 10th day, this increase was found in *RpAtg8* knockdown insects compared to control insects. The balance between lipolysis and lipogenesis controls lipid storage homeostasis and LD dynamics (Olzmann and Carvalho, 2019). Therefore, larger LDs are associated with lower lipid mobilization activity, although no differences in TAG content were detected. Alternatively, changes in the profile of LDs may occur to redirect the LDs to another degradative pathway, as previously described in mouse hepatocytes, in which LDs were preferentially directed based on their size, with the largest being directed to lipolysis (Schott et al., 2019). In addition, LDs are very dynamic organelles and can vary greatly in terms of number, size and cellular distribution. They can grow through fusion, lipid synthesis or the transfer of stored lipids, and these events can be affected by metabolic signals as well as their protein and total lipid compositions (Krahmer et al., 2013; Thiam and Beller, 2017). However, the coordination of these events is still poorly understood. For instance, it is possible that changes in the composition of the phospholipid monolayer after *RpAtg6* and *RpAtg8* knockdown may have affected the biophysical properties of the LDs, which resulted in their fusion and increased size. Of course, this is very speculative, and additional studies are necessary to clarify how the LD population changes during the reproductive cycle in this insect.

The general physiology of individuals can be altered at the molecular level by changes in environmental factors (temperature, humidity, photoperiod, etc.), nutritional status (different cycles and types of diet), and immunological stress (exposure to pathogens, symbionts, etc.), among many other factors (Schmidt-Nielsen, 1997; Hoffmann, 2012; Zhang et al., 2019). As we observed only moderate phenotypes in terms of lipophagic activation, we hypothesized that, in environments different from our usual insect care facilities, lipid targeting by autophagy under fed conditions might be necessary. Our insects are maintained in carefully regulated ideal environmental conditions and do not experience challenges such as infections, predatory species, and fluctuations in temperature or humidity. Therefore, we cannot rule out the possibility that lipophagy activity emerges as needed after insects are exposed to environmental changes, allowing adjustments to novel situations. To examine this possibility, we tested the lipid-targeting activity of autophagy under conditions of forced flight exercise, which does not occur under general in-house conditions (our insects do not usually fly in their containers), and the mobilization of internal stores is mainly required. In *R. prolixus*, as well as in other insects, lipids are the primary fuel for flight. The energy provided for this activity originates from the TAG already present in the flight muscle (Ward et al., 1982) and from lipids stored in the fat body, which are delivered to the flight muscle through the lipophorin transport system (Oliveira et al., 2006). The experiments showed that the forced flight capacity was significantly reduced in the knockdown females. Therefore, we hypothesize that the use of energy reserves in these insects could be reduced. Although individual variation was high and statistically significant differences were not detected in all cases, differences in TAG and protein accumulation were observed after flight activity in the knockdown samples, which suggests a difficulty in using these reserves during forced exercise and specific activation of autophagy directed toward the mobilization of energy stores. By directing TAG for degradation in the lysosome, lipophagy contributes to the generation of fatty acids, which can be used by mitochondria for the production of ATP (Zechner et al., 2017). Thus, our findings that ATP levels were reduced in the flight muscle of knockdown insects after the flight period further support the hypothesis that reduced autophagy impaired TAG mobilization, resulting in reduced ATP levels in those cells.

Upon comparing the *RpAtg6* and *RpAtg8* knockdown phenotypes observed in the fed state with those observed during starvation (Santos-Araujo et al., 2020), we discovered that while the LD diameters increased in both scenarios, the storage of TAG in the fat body was not as impacted in the fed state as it was during starvation. Nonetheless, although a reduction in forced flight activity was observed in both conditions, the knockdowns in the fed state had a greater impact on the TAG stores in the flight muscle. Notably, different enzymes involved in lipid metabolism were affected in the fat body under both conditions. In the fed state, ACC and DGAT2 were downregulated, and ACSL2, CPT1, and Bmm were upregulated; however, during starvation, Bmm was downregulated, and AKHr was upregulated. Taken together, these data indicate that autophagy and lipid metabolism are differentially regulated under the two distinct dietary conditions, highlighting the intricate physiological adaptations developed by this insect to cope with different conditions.

In conclusion, our investigation highlights the multifaceted role of autophagy in lipid metabolism and its broader physiological implications in insects. The relationship between autophagy and lipid metabolism is complex and dynamic and is influenced by various factors, including environmental conditions, nutritional status, and physiological demands. While our findings suggest that autophagy is not a primary driver of lipid mobilization in the fed state under typical laboratory conditions, the results underscore the remarkable adaptability of cellular processes. Furthermore, the observed alterations in LD dynamics and gene expression profiles in response to autophagy disruption suggest the presence of intricate regulatory mechanisms governing energy homeostasis and cellular maintenance. The potential role of lipophagy in facilitating energy mobilization during high-performance activities, such as flight, is particularly intriguing and provides new opportunities for determining the adaptability of insects in response to changing environmental and physiological demands. Our study underscores the need for further exploring the nuanced functions of autophagy in diverse contexts, shedding light on its evolutionary significance and potential as a target for pest control and disease vector management strategies.

## Data Availability

The original contributions presented in the study are included in the article/Supplementary material, further inquiries can be directed to the corresponding author.
