# Supplementary material for: In the fed state, autophagy plays a crucial role in assisting the insect vector Rhodnius prolixus mobilize TAG reserves under forced flight activity
Source: Front Physiol. 2024 Apr 25;15:1352766. doi: 10.3389/fphys.2024.1352766 (PMC11079428; doi:10.3389/fphys.2024.1352766)
Supplement: Supplementary file 1 [file Table1.DOCX]

Table S1: Primer sequences used for qPCR amplification

| Gene | Primer sequence | |  |
| --- | --- | --- | --- |
|  | Forward | Reverse | Reference |
| *RpAtg6* | CCGCTCCTGTAGACTGGTC | GCCACCATCGCAGCATCAAATTTTG | (Vieira et al., 2018) |
| *RpAtg8* | GAACAATGTAATCCCACCGACAA | CCATAGACATTTTCATCACTATACG | (Pereira et al., 2020) |
| *Rp18S* | TCGGCCAACAAAAGTACACA | TGTCGGTGTAACTGGCATGT | (Majerowicz et al., 2011) |
| *RpCPT-1* | AAACACCACATGGCCAAACT | GAAACGCCGTATCCATCATC | (Alves-Bezerra et al., 2016b) |
| *RpGPAT-1*  *RpGPAT-4* | TTGTCTGCGACGAACAAGGA  GGGCGATTGTTTGCGATGTA | AACCGTCGGGTTGCTTCTCT  ACGGCTTTAACCCTGTTAGCAA | (Alves-Bezerra and Gondim, 2012)  (Alves-Bezerra et al., 2017) |
| *RpACSL-2* | TAGCCGTAATGGCAGAACGC | CCATGGGCAGCTAATTCTGC | (Alves-Bezerra et al., 2016b) |
| *RpAKHr* | TTCTATTCGCATGCACCAAC | ACTAGTGCGCGAGTTGTTTG | (Alves-Bezerra et al., 2016a) |
| *RpBmm*  *RpACC*  *RpDGAT1*  *RpDGAT2*  *BiP1*  *BiP2*  *Bip3*  *BiP4*  *BiP5*  *PDI2*  *PDI4*  *PDI5* | ACATGGATGGAGGATTCAGTG  TGGGCTGGAACCGTAGTTGCG  TCACAACCGGATAAACCTTG  CCGGCTCACTTACTTACAAC  GAGCGCAACGCTAGAATACC  GGTATCCCCCAGATTGAGGT  AGGTGGTGGAACCTTTGATG  CTTATGGAGCTGCTGTGCAA  CTAACACCGGCAACACCTTT  TCACTCTTGCCAAGGTCGAT  TTTCACTGGAGGCCTAGACG  GGAATCAATCTGGACCGAGT | GGGCATATATCGGTTTCACC  TGCGGGATCGGCTGGAAGTTGT  TGAGAGCCAGTCAACACTAT  CGATTGGCTTTCCAACTACA  TGGCATCCAGATCGAATGTA  TGCGTTTCTAGCATTGATCG  GATGATAATGCTCGCTTGGC  CTCCGGGTTGGTTATCTGAA  ACTCTCCGCTGTTTCCTTCA  TTCGCACTTGCACCAACTTG  GGATAGAGTGCAGCCGTTTG  CGCACAACAGATGGAGTAGC | (Majerowicz et al., 2016)  (Saraiva et al., 2021)  Not annotated  Not annotated  (Rios et al., 2021)  (Rios et al., 2021)  (Rios et al., 2021)  (Rios et al., 2021)  (Rios et al., 2021)  (Rios et al., 2021)  (Rios et al., 2021)  (Rios et al., 2021) |

AKHr, Adipokinetic hormone receptor; Bmm, Brummer lipase; CPT1, Carnitine palmitoyltransferase I; GPAT1, Glycerol-3-phosphate acyltransferase 1; GPAT4, Glycerol-3-phosphate acyltransferase 4; ACSL2, Long-chain acyl-CoA synthetase 2; ACC, Acetyl-CoA carboxylase; DGAT1, Diacylglycerol O-acyltransferase 1; DGAT2, Diacylglycerol O-acyltransferase 2; BiP, Immunoglobulin binding protein; PDI, Protein disulfide isomerase.

Table S2: Primer sequences used as template for dsRNA synthesis (PCR)

| Gene | Primer sequence | |  |
| --- | --- | --- | --- |
|  | Forward | Reverse | Reference |
| *RpAtg6* | TAATACGACTCACTATAGGGTACT  GCAGTTTGGGAGAACATACTCTCG | TAATACGACTCACTATAGGGTACT  CTGTACACTTCTGTGTTCATCTTCC | Vieira et al., 2018 |
| *RpAtg8* | TAATACGACTCACTATAGGGTACT  ATGAAGTTTCAATATAAAGAAGAGC | TAATACGACTCACTATAGGGTACT  ATCTTCTTCATGATGTTCCTGAT | Pereira et al., 2020 |

The T7 promoter sequence is underlined.

Alves-Bezerra, M., De Paula, I. F., Medina, J. M., Silva-Oliveira, G., Medeiros, J. S., Gäde, G., et al. (2016a). Adipokinetic hormone receptor gene identification and its role in triacylglycerol metabolism in the blood-sucking insect Rhodnius prolixus. *Insect Biochem Mol Biol* 69, 51–60. doi: 10.1016/j.ibmb.2015.06.013.

Alves-Bezerra, M., and Gondim, K. C. (2012). Triacylglycerol biosynthesis occurs via the glycerol-3-phosphate pathway in the insect Rhodnius prolixus. *BBA - Molecular and Cell Biology of Lipids* 1821, 1462–1471. doi: 10.1016/j.bbalip.2012.08.002.

Alves-Bezerra, M., Klett, E. L., De Paula, I. F., Ramos, I. B., Coleman, R. A., and Gondim, K. C. (2016b). Long-chain acyl-CoA synthetase 2 knockdown leads to decreased fatty acid oxidation in fat body and reduced reproductive capacity in the insect Rhodnius prolixus. *Biochim Biophys Acta Mol Cell Biol Lipids* 1861, 650–662. doi: 10.1016/j.bbalip.2016.04.007.

Alves-Bezerra, M., Ramos, I. B., De Paula, I. F., Maya-Monteiro, C. M., Klett, E. L., Coleman, R. A., et al. (2017). Deficiency of glycerol-3-phosphate acyltransferase 1 decreases triacylglycerol storage and induces fatty acid oxidation in insect fat body. *Biochim Biophys Acta Mol Cell Biol Lipids* 1862, 324–336. doi: 10.1016/j.bbalip.2016.12.004.

Majerowicz, D., Alves-Bezerra, M., Logullo, R., Fonseca-De-Souza, A. L., Meyer-Fernandes, J. R., Braz, G. R. C., et al. (2011). Looking for reference genes for real-time quantitative PCR experiments in Rhodnius prolixus (Hemiptera: Reduviidae). *Insect Mol Biol* 20, 713–722. doi: 10.1111/j.1365-2583.2011.01101.x.

Majerowicz, D., Hannibal-Bach, H. K., Castro, R. S. C., Bozaquel-Morais, B. L., Alves-Bezerra, M., Grillo, L. A. M., et al. (2016). The ACBP gene family in Rhodnius prolixus: Expression, characterization and function of RpACBP-1. *Insect Biochem Mol Biol* 72, 41–52.

Pereira, J., Diogo, C., Fonseca, A., Bomfim, L., Cardoso, P., Santos, A., et al. (2020). Silencing of RpATG8 impairs the biogenesis of maternal autophagosomes in vitellogenic oocytes, but does not interrupt follicular atresia in the insect vector Rhodnius prolixus. *PLoS Negl Trop Dis* 14, e0008012. doi: 10.1371/journal.pntd.0008012.

Rios, T., Bomfim, L., and Ramos, I. (2021). The transition from vitellogenesis to choriogenesis triggers the downregulation of the UPR sensors IRE1 and PERK and alterations in the ER architecture in the follicle cells of the vector Rhodnius prolixus. *Cell Tissue Res*. doi: 10.1007/s00441-021-03547-z.

Saraiva, F. B., Alves-Bezerra, M., Majerowicz, D., Paes-Vieira, L., Braz, V., Almeida, M. G. M. D., et al. (2021). Blood meal drives de novo lipogenesis in the fat body of Rhodnius prolixus. *Insect Biochem Mol Biol* 133. doi: 10.1016/j.ibmb.2020.103511.

Vieira, P. H., Bomfim, L., Atella, G. C., Masuda, H., and Ramos, I. (2018). Silencing of RpATG6 impaired the yolk accumulation and the biogenesis of the yolk  organelles in the insect vector R. prolixus. *PLoS Negl Trop Dis* 12, e0006507. doi: 10.1371/journal.pntd.0006507.
