# Supplementary material for: In the fed state, autophagy plays a crucial role in assisting the insect vector Rhodnius prolixus mobilize TAG reserves under forced flight activity
Source: Front Physiol. 2024 Apr 25;15:1352766. doi: 10.3389/fphys.2024.1352766 (PMC11079428; doi:10.3389/fphys.2024.1352766)
Supplement: Supplementary file 2 [file Image1.pdf]

Santos-Araujo et al., Supplementary material:

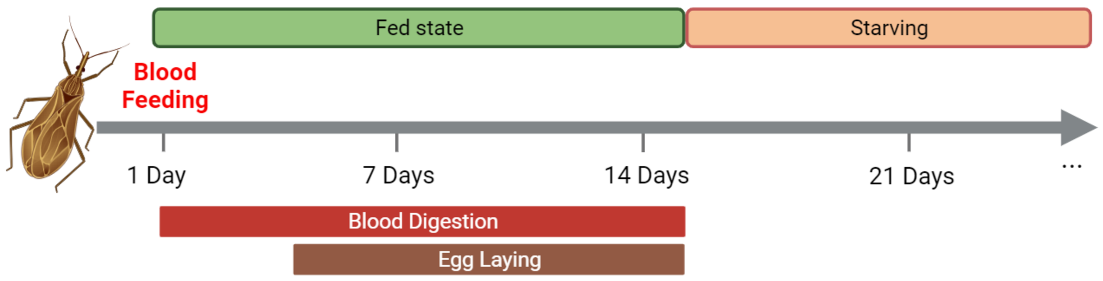

Figure S1: Nutritional states of *R. prolixus* over our insectarium feeding cycle. In our insect care facilities, the adult insects are blood-fed every 21 days. Fully gorged adult females finalize blood digestion and stop laying eggs around day 15-17 after blood feeding (Coelho et al., 1997).
